# Notes on Preparations for the Sick

**Published:** 1908-12

**Authors:** 


### Notes on Preparations for the Sick.

**Sauerin Tablets.**—ALLEN AND HANBURYS, London.—These are a culture of vigorous strains of the bacillus acidi lactici, in tablet form, for internal use, or for the production of sour or curdled milk.

The researches of Metchnikoff and other observers have shown that an active strain of the lactic acid-producing bacterium, taken with a suitable medium for its multiplication—such as milk or sugary substances—passes through the stomach and establishes itself in the large intestine, where, by the production of lactic acid, it inhibits the growth of the Bacillus coli communis and other intestinal bacteria.

Attention has therefore been directed to the use of these organisms in pure culture, in disorders arising from absorption of toxins, and as a general intestinal disinfectant.

One or two tablets should be taken three or four times daily after meals.

---

**Lactéol Tablets.**—ROBERTS & CO., London.—These are said to be a most efficacious means of administrating lactic acid bacilli. They are prepared by a process which ensures the continued vitality of the bacilli, they are tasteless, and have no putrefactive odour.

When decomposing matter is inoculated with lactic ferments the putrefactive process is arrested, and the place of the putrefactive organisms is taken by the lactic bacilli. This is the way lactéol acts in the intestine where the pathogenic bacteria are gradually replaced by lactic bacilli and normal intestinal organisms.

In various intestinal conditions, where intestinal antisepsis is desired, it is stated that three to five tablets daily, dissolved in a little boiled or sweetened water, give good results.

**Lacto - Bacteria Tablets.**—STAUFFER, 12, 13 Cullum Street, London, E.C.—These tablets, containing 30 centigrammes each, have been manufactured from the constituents of milk by pure cultures of lactic acid ferments. They are believed to prevent or moderate the putrefactive fermentation in the bowels, and are said to give much better results than the ordinary antiseptic drugs used for this purpose. They are almost tasteless, and may either be swallowed like a pill, or first masticated, or they may be dissolved in milk. The usual dose is three to four in the day to be taken after meals.

---

**Junket Tablets.**—CHR. HANSEN, Copenhagen; 77 St. Thomas Street, London, S.E.—We have found these tablets very efficacious for the production of junket, and hence they are being found useful wherever a milk food is desirable, particularly in chronic kidney troubles. They contain nothing but the pure rennet ferment from the stomach of the calf.

---

**Sterilla Soap.**—MATTHEWS, Clifton.—We have made further experiments with Sterilla, a trade name given to a liquid soap produced locally. It deserves mention, perhaps, because it is a solution of potash soap in one of the naphthol series, and therefore costs less to produce than the ordinary alcohol-ether soap (*vide* page 284).

We have made some experiments to determine whether it satisfactorily replaces the alcohol-ether soap. It does so, and is much more economical than the alcohol solution. As far as laboratory tests go, a solution of Sterilla, 1 in 20, is equal to 1 in 20 carbolic acid solution, so that it has some antiseptic properties also. But it must be remembered that no mere washing with soap is sufficient to completely sterilise skin. While Sterilla soap kills laboratory strains of diphtheria and typhoid bacilli when these organisms are placed under the nails and the parts are scrubbed well, it does not remove the staphylococcus epidermidis albus; neither does biniodide of mercury soap, for the matter of that, although it partially removes the skin organisms, and thus is more efficient. But biniodide soap cannot be entirely depended upon; the cocci are never entirely killed. Subsequent immersion in disinfectant solution is necessary.

The undiluted Sterilla solution is less effective than the 1 in 20. It is advisable to employ a 1 in 10 solution whenever organic material is to be disinfected, and to regard the soap as an excellent preparer of the skin for final immersion in a standard solution of sterilla, or preferably of another disinfectant. The simple washing with Sterilla, in our experiments, did not replace the immersion in some antiseptic solution. It leaves the skin in a better condition than does the biniodide, and is of course more useful for the disinfecting of instruments.

The 1 in 10 solution, placed on mirrors and instruments, killed diphtheria and cocci in one minute.

---

**Casona: A new form of Cheese.**—SEVERN VALLEY DAIRY PRODUCTS CO., Stonehouse, Glos.—Cheese as a food has become of late years quite an important part of the dietetic programme of the supposed invalid. Haig's uric acid theories, the modern views on purin bodies, and the necessity of finding varieties of suitable food for the diabetic, has given a great impetus to the production and use of dairy products of various kinds. One of the latest of these is that which has been designed, with the assistance of Dr. Francis T. Bond, more especially for the diabetic. Several varieties are made: cream, plain, anchovy, savoury, piquant, and celery.

They should be a valuable addition to the dietary of those who like the last new thing, or those who are obliged to refrain from the old and familiar methods of feeding which have long been in vogue, but do not suit all patients alike.

---

**Ceridin: The Active Principles of Yeast** (*vide* page 285).—BOEHRINGER & SOEHNE, Mannheim.—We find that the Ceridin tablets are for children only: for adults the drug is put up in the form of pills containing 1.5 grs. of Ceridin. Further reports of a favourable character have been received. It has been found to cut short attacks of furunculosis and acne, and is also of use by regulating the action of the bowels in milder forms of constipation.

